# External validation of a multimodality deep-learning normal tissue complication probability model for mandibular osteoradionecrosis trained on 3D radiation distribution maps and clinical variables

**DOI:** 10.1016/j.phro.2024.100668

**Published:** 2024-11-02

**Authors:** Laia Humbert-Vidan, Christian R. Hansen, Vinod Patel, Jørgen Johansen, Andrew P. King, Teresa Guerrero Urbano

**Affiliations:** a Department of Medical Physics, Guy’s and St Thomas’ NHS Foundation Trust, London, UK; b School of Cancer and Pharmaceutical Sciences, Comprehensive Cancer Centre, King’s College London, London, UK; c Department of Clinical Research, University of Southern Denmark, Odense, Denmark; d Laboratory of Radiation Physics, Odense University Hospital, Odense, Denmark; e Department of Oral Surgery, Guy’s and St Thomas’ NHS Foundation Trust, London, UK; f Department of Oncology, Odense University Hospital, Odense, Denmark; g School of Biomedical Engineering and Imaging Sciences, King’s College London, London, UK; h Department of Clinical Oncology, Guy’s and St Thomas’ NHS Foundation Trust, London, UK

**Keywords:** Osteoradionecrosis, Deep learning, Radiotherapy, Toxicity, Head and neck, NTCP, Multimodality

## Abstract

**Background and purpose:**

While the inclusion of spatial dose information in deep learning (DL)-based normal-tissue complication probability (NTCP) models has been the focus of recent research studies, external validation is still lacking. This study aimed to externally validate a DL-based NTCP model for mandibular osteoradionecrosis (ORN) trained on 3D radiation dose distribution maps and clinical variables.

**Methods and materials:**

A 3D DenseNet-40 convolutional neural network (3D-mDN40) was trained on clinical and radiation dose distribution maps on a retrospective class-balanced matched cohort of 184 subjects. A second model (3D-DN40) was trained on dose maps only and both DL models were compared to a logistic regression (LR) model trained on DVH metrics and clinical variables. All models were externally validated by means of their discriminative ability and calibration on an independent dataset of 82 subjects.

**Results:**

No significant difference in performance was observed between models. In internal validation, these exhibited similar Brier scores around 0.2, Log Loss values of 0.6–0.7 and ROC AUC values around 0.7 (internal) and 0.6 (external). Differences in clinical variable distributions and their effect sizes were observed between internal and external cohorts, such as smoking status (0.6 vs. 0.1) and chemotherapy (0.1 vs. −0.5), respectively.

**Conclusion:**

To our knowledge, this is the first study to externally validate a multimodality DL-based ORN NTCP model. Utilising mandible dose distribution maps, these models show promise for enhancing spatial risk assessment and guiding dental and oncological decision-making, though further research is essential to address overfitting and domain shift for reliable clinical use.

## 1 Introduction

Mandibular osteoradionecrosis (ORN) is a severe late side effect that affects 4–15 % [1] of patients who have undergone radiation therapy (RT) for head and neck cancer. Radiation-induced fibrosis of the irradiated tissues extends to the blood vessel walls, eventually resulting in a reduced blood supply and subsequent necrosis of the lower jawbone [2]. Depending on the severity, the clinical management of ORN may range from more conservative treatments to complex and costly surgical interventions such as segmental resection of the mandible, which are highly detrimental to the patient’s quality of life [3].

In addition to radiation dose, other risk factors have been identified, including dental extractions, pre-RT surgery to the mandible, smoking, poor oral hygiene and a sub-optimal dentition, and multiple studies [4], [5], [6], [7], [8] have analysed associations between these factors and the development of mandibular ORN. An increased incidence of ORN has also been observed in the HPV-associated OPC group of patients that are typically younger, with better dental status and without the lifestyle factors associated with ORN (e.g. smoking and alcohol) [5].

In van Dijk et al. [9] the first ORN normal tissue complication probability (NTCP) model was developed based on the dose to 30 % of the mandible bone (D_30%_) and pre-RT dental extractions as predictors. Dose-volume parameters have been used clinically and in NTCP models for decades, but they have limitations [10], [11]. With such parameters, the volumetric dose distribution within an organ is reduced to a unidimensional number that does not capture potential clinically relevant spatial information. The dose-volume histogram (DVH) effectively, albeit incorrectly, assumes a spatially invariant dose–effect relationship within a structure. As a result, a DVH-based NTCP model might not correctly reflect the true relationship between radiation dose and toxicity for each organ sub-unit. Moreover, anatomically relevant spatial information is not captured.

The use of radiation dose distribution maps as the dose information for predicting radiation-induced toxicities has been explored with deep learning (DL) methods [12], [13]. More recently, this approach has also been introduced in the prediction of mandibular ORN and compared to DVH-based approaches [14], [15]. However, the DL models in these studies were trained on single modality data (image-based dosimetric information) and did not include clinical (non-imaging) variables. Based on when the fusion of the two data types takes place, there are different DL multimodality data fusion strategies: Early Fusion, Joint Fusion or Late Fusion [16], [17]. Moreover, these studies featured only internal validation on a holdout subset of the data used for training. While internal validation may inform on the model performance during its development, the external validation of a model will provide insight into how well a model may be applicable to independent datasets, which is a crucial step for translating from the research to the clinical setting [18], [19]. It also provides insight into the model’s limitations and potential directions for further development. This is particularly relevant when using DL methods, which are less interpretable than more traditional statistical approaches. While external validation studies of traditional DVH-based NTCP models exist [20], [21], [22], model validation efforts in the context of spatial dose NTCP modelling are limited, probably due to the technical complexities involved in the data preparation process. However, external validation of such models is necessary for their acceptance in a clinical context largely dominated by DVH-based models. The current study aimed to externally validate a DL-based ORN NTCP model trained on 3D radiation dose distribution maps and clinical variables.

## 2 Materials & methods

### 2.1 Patient selection

The model was developed with a dataset of 92 ORN cases and 92 controls from a UK population treated at Guy’s and St Thomas’ Hospitals (GSTT) between 2011 and 2022 under ethics approval from the North West- Haydock Research Ethics Committee of the NHS Health Research Authority (REC reference 18/NW/0297, IRAS project ID: 231443). The validation dataset consisted of 41 ORN cases and 41 controls from an independent dataset from a Danish population treated at Odense University Hospital (OUH) between 2007 and 2015 [4]. Data extraction of clinical and treatment-related parameters, as well as data handling, was approved by The Danish Data Protection Agency (Jr. 16/29136) and The Danish Patient Safety Authority (Jr. 3-3013-1798/1/). In both cohorts, the subjects were retrospectively selected from clinical databases using a control-case matching approach based on the primary tumour site – oropharynx, oral cavity, larynx and others (paranasal sinus, salivary glands and unknown primary) – and treatment year (Supplement A).

### 2.2 Treatment

All subjects in both cohorts were treated with curative intent using intensity-modulated radiation therapy (IMRT). The subjects in the GSTT cohort were planned with the Monaco (Elekta AB, Stockholm, Sweden) and Eclipse (Varian Medical Systems, Milpitas, CA) treatment planning systems (TPS); Supplement B describes how the dosimetric differences from the two TPS were addressed. Radical primary IMRT cases were prescribed a total dose of 65–70 Gy in 30–35 fractions and 55 Gy in 20 fractions in selected cases. Radical post-operative IMRT (PORT) cases were prescribed 60–66 Gy in 30–33 fractions and 50 Gy in 20 fractions in selected cases. The subjects in the OUH cohort were planned on the Pinnacle TPS (Philips Radiation Oncology Systems, Fitchburg, WI). The dose prescription was 66 or 68 Gy in 2 Gy fractions 5–6 fractions per week following the Danish Head and Neck Cancer Group (DAHANCA) national guidelines from 2013 [23], [24]. Patients from the OUH cohort were treated with simultaneous integrated boost, i.e., 60 Gy to CTV2 and 50 Gy to CTV3 (elective regions) and concomitant weekly cisplatin and radiosensitiser nimorazole were prescribed according to the DAHANCA guidelines.

### 2.3 Clinical data

Demographic and clinical data was collected from clinical and treatment records in the MOSAIQ and Eclipse systems (GSTT) and the Pinnacle system (OUH) for the variables described in Table 1. No data imputation was performed as only subjects with complete datasets were included. With regards to the NTCP endpoint, mandibular ORN severity was staged according to the Notani classification system [25] in both institutions. However, since the prediction model was developed as a binary classification model, the ORN stage was dichotomised, and any grade of ORN was considered an ORN case.

**Table 1:** Clinical data distribution for the internal and external datasets. Statistical significance of difference between groups is provided by the Mann-Whitney *U* test p-value and the Cohen’s d value indicates the effect size.

	Internal dataset			External dataset		
	**ORN+ / ORN-**	**p**	**d**	**ORN+ / ORN-**	**p**	**d**
Variable	N = 92 / 92			N = 41 / 41		
Primary site						
− Oral cavity	28 (30 %) / 28 (30 %)			19 (46 %) / 16 (39 %)		
− Oropharynx	52 (57 %) / 52 (57 %)			21 (51 %) / 25 (61 %)		
− Larynx	3 (3 %) / 3 (3 %)			1 (2 %) / 0 (0 %)		
− Other*	6 (10 %) / 6 (10 %)			0 (0 %) / 0 (0 %)		
Age (median, IQR)	62 (13) / 61 (15)	0.46	0.1	59 (12) / 60 (13)	0.42	−0.2
Gender (male)	66 (72 %) / 72 (78 %)	0.40	0.2	30 (73 %) / 31 (76 %)	1.00	0.1
Smoking	47 (51 %) / 21 (23 %)	<0.001	0.6	25 (61 %) / 24 (59 %)	1.00	0.1
Pre-RT extractions	55 (60 %) / 50 (61 %)	0.55	0.1	34 (83 %) / 30 (73 %)	0.42	0.2
PORT	35 (38 %) / 35 (38 %)	1.00	0.0	16 (39 %) / 13 (32 %)	0.64	0.2
Chemotherapy	59 (64 %) / 57 (62 %)	0.88	0.1	7 (17 %) / 15 (37 %)	0.08	−0.5

*Other: paranasal sinus, salivary glands, unknown primary.

### 2.4 Dosimetric data

The mandibles were segmented on the radiotherapy planning computed tomography (CT) images by each centre on their own cohort and using their respective TPS. The entire mandible structure was contoured, including the condyles and excluding the teeth. The reconstructed bone was not included in cases where mandibulectomy and subsequent mandible reconstruction were performed. For the training dataset, the RT dose, CT image and RT structure DICOM files, as well as dose-volume histogram data – DVH, were exported from the TPS. The external validation dataset was extracted from the Danish RT doseplan database, the DICOM Collaboration system (DcmCollab) [26]. The mandible 3D dose distribution maps were created by masking the clinically planned radiation dose distribution volume with the manually contoured mandible structure. Dose maps were normalised to values between 0 and 1 using global minimum and maximum intensities across the entire dataset. Correction to an equivalent dose in 2 Gy fractions (EQD2) was applied to the dose maps with an alpha–beta ratio of 3 Gy for late effects [27]. Image processing was performed using 3D Slicer and ITK-Snap software and included the steps described in [28].

### 2.5 ORN prediction models

**Fig. 1:**
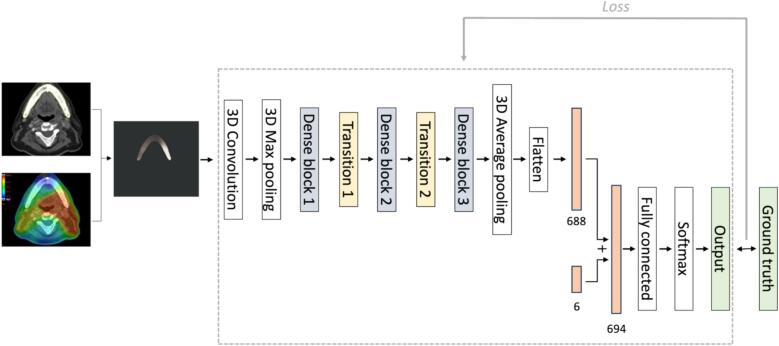
Deep learning workflow and architecture of the 3D-mDN40 model. The clinical variables (6) were concatenated with the image features (688) extracted from the mandible dose maps into a single feature vector (694,1) before a final fully connected layer consisting of 64 hidden neurons and two output channels. A final softmax layer was added to obtain the final class probabilities. Mandible dose maps were computed from the planned radiation dose distribution maps and the manually contoured mandible structure.

In previous work [28], a single modality 3D-DN40 CNN was trained with only dose maps; this model was retrained on the updated dataset (GSTT) used in this study. Additionally, a multimodality 3D DenseNet-40 (3D-mDN40) convolutional neural network (CNN) was trained on 3D radiation dose distribution maps of the mandible and clinical variables for the binary classification of ORN vs. no ORN subjects using the internal cohort. A 3D-DN40 CNN is a shallower version of the more widely used DenseNet CNN [29]. It consists of three dense blocks and two transition blocks, where all the convolutional, pooling, batch normalisation and dropout operations are three-dimensional. Each dense block is formed of 12 dense layers. The output from the last dense block is reduced to one dimension with a 3D average pooling layer and flattened. Finally, a fully connected layer followed by a softmax layer provides the binary classification probabilities. The 3D-mDN40 architecture implemented (Fig. 1) followed a Joint Fusion approach [17], which inherited from the 3D-DN40 CNN. The code is available in https://github.com/KCLMMAG/LaiaHumbertVidan.git. The 3D-mDN40 and 3D-DN40 models were implemented within the Medical Open Network for Artificial Intelligence (MONAI) (https://monai.io/) Pytorch-based framework. The Adam optimisation algorithm and the categorical cross entropy loss function (torch.nn.CrossEntropyLoss) were used. A hyperparameter grid search was performed which included the following hyperparameters and values: dropout 0.6, 0.8; learning rate 0.01, 0.001, 0.0001; batch size 10, 16; weight decay 0.01, 0.001, 0001; epochs 50, 100, 300. Small 3D random rotation (−0.1 to 0.1 rad) and zoom (0.8 to 1.2) data augmentations were applied to the dose maps of the training set.

For completeness, the 3D-DN40 and 3D-mDN40 models were compared to a logistic regression (LR) stepwise forward selection model trained on clinical variables and DVH metrics (D2%, D5%-D95%, D98%, V5Gy-V70Gy) with prior correlation-based feature pre-selection (Spearman coefficient > 0.8).

### 2.6 Statistical analysis

The 3D-mDN40, 3D-DN40 and LR models were internally and externally validated. In both validation processes, the models’ performance was assessed in terms of their discriminative ability and calibration. Analysis was performed as per the TRIPOD + AI reporting guidelines [19] (see Supplement C).

**Fig. 2:**
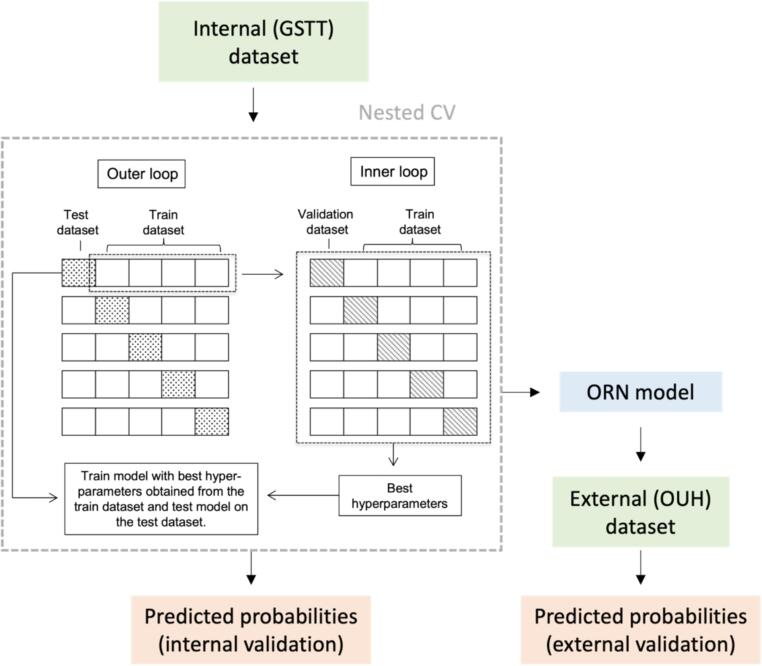
Model training workflow. For each of the outer CV folds, hyperparameter optimisation is performed j times in an inner j-fold CV approach where the outer CV train dataset is further split into train and validation sets. Finally, for each of the outer CV folds, the entire training set is used for training using the optimised hyperparameters obtained from the inner CV, and the prediction accuracy can be calculated on the held-out test set. In this way, the test set of each outer CV fold remains completely unseen, avoiding the bias introduced in traditional CV. A 5-fold CV approach is stratified when the class balance is maintained in all CV folds.

For the DL models, internal validation was performed using a stratified nested 5-fold cross-validation (CV) approach, which consists of an inner CV loop encompassed by an outer CV loop (Fig. 2). The external validation process indicates how well the trained DL model generalises on an independent dataset, i.e. whether the model can produce predicted probabilities that are accurate and well-calibrated not just on the training data but also on unseen data. External validation was performed by testing the discrimination and calibration of the model on the external (independent) dataset.

Model discrimination was measured using the accuracy, recall, specificity, precision and area under the receiver operating characteristic curve (AUROC) metrics. The ROC curve was obtained by plotting the predicted probabilities for the positive class (ORN). Model classification performance metrics considered include accuracy, recall, specificity, precision and F1 score; to calculate these, the probability classification threshold was set to 0.5.

Model calibration, i.e. alignment between the predicted probabilities and the actual class (i.e. ORN vs. no ORN), was measured with the logarithmic loss (LogLoss). The Brier score (BS) was used to assess the overall model performance measure. Log Loss and Brier score values may range between 0 and 1, and lower values correspond to a better calibration.

Univariable significance of the clinical and demographic variables was calculated with the Mann-Whitney U and Chi-squared statistical tests for the continuous and categorical variables, respectively, with the significance level set to 0.05. Effect size is reported by the Cohen’s d measure to define small (0.2), moderate (0.5) or large (0.8) effects for a given variable, with its sign (positive/negative) indicating the direction of the effect.

## 3 Results

### 3.1 Clinical and demographic data

The clinical and demographic data distributions for the internal and external datasets are described in Table 1. The mean follow-up time for the internal cohort was 3.6 (0.1–6.6) and 4.2 (0.4–7.7) years for the ORN and control groups, respectively. The mean time to ORN from the end of RT was 1.5 (0.0–7.3) years. The mean follow-up time for the external cohort was 3.7 (0.3–9.1) and 2.9 (0.2–8.0) years for the ORN and control groups, respectively. The mean time to ORN was 1.3 (0.0–7.6) years.

### 3.2 Model performance

**Fig. 3:**
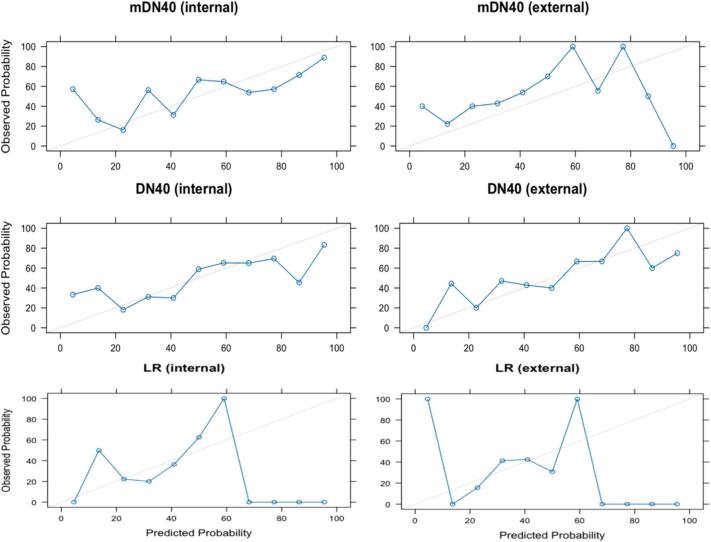
Calibration curves of the 3D-mDN40 (top), 3D-DN40 (middle) and LR (bottom) models at internal (left) and external (right) validation. The x-axis represents the mean predicted probability of the positive outcome for each bin. The y-axis represents the fraction of true positives (i.e., ORN) within each bin. The grey diagonal line represents the ideal calibration curve (i.e., perfect alignment).

Despite the larger F1-score observed for the LR model (with a 0.5 classification threshold), no significant difference in discriminative performance was observed between the 3D-mDN40, 3D-DN40 and LR models (see Table 2, Fig. 3 and supplementary Fig. D1) at internal or external validation. DeLong p-values across all model pair comparisons ranged between 0.84 and 0.96 and between 0.46 and 0.97 at internal and external validation, respectively. Despite lower Log loss values, indicating more confident predictions, calibration curves for the LR model displayed a poorer alignment between the observed and predicted probabilities.

**Table 2:** Model calibration and discrimination performance results at internal and external validation of the 3D-DN40, 3D-mDN40 and LR models. The classification threshold for the predicted probabilities was set to 0.5 to calculate the classification performance metrics.

	3D-DN40		3D-mDN40		LR	
	Internal	External	Internal	External	Internal	External
Brier score	0.23	0.24	0.23	0.26	0.22	0.21
Log loss	0.66	0.70	0.72	0.74	0.63	0.61
AUROC (95 % CI)	0.69 (0.62–0.77)	0.66 (0.54–0.78)	0.70 (0.62–0.78)	0.66 (0.54–0.78)	0.69 (0.52–0.86)	0.64 (0.54–0.74)
Accuracy	0.67	0.61	0.66	0.57	0.69	0.68
Recall	0.63	0.73	0.72	0.85	1.0	1.0
Specificity	0.71	0.49	0.61	0.29	0.16	0.02
Precision	0.68	0.59	0.65	0.55	0.67	0.68
F1 score	0.66	0.65	0.68	0.67	0.80	0.81

## 4 Discussion

In this study we have externally validated a multimodality DL-based ORN NTCP model trained on 3D radiation dose distribution maps and clinical data. In previous work [28], we showed that dose map-based ORN incidence predictions were comparable to DVH-based predictions. In the present study, we have confirmed this previous finding and have expanded from a single modality model to a multimodality DL-based ORN NTCP model including clinical variables alongside radiation dose distribution maps and externally validated both models.

While the performance difference between the single modality (3D-DN40) and multimodality (3D-mDN40) models was not statistically significant, the inclusion of clinical variables using a joint fusion approach resulted in slightly worse external validation results. The explanation for this is possibly two-fold: increased overfitting due to added parameters in the multimodality model and a domain shift between the two independent datasets. With regards to the dosimetric information, radiation doses in the mandible were lower overall in the external dataset than in the internal dataset. Interestingly, however, the tails of the DVH, doses at the higher dose points, were lower for the internal DVH, and the average internal control doses were similar to the average external ORN doses (Supplement E). On the other hand, there were large differences in clinical variable distribution between the two datasets for some variables (Table 1). Chemotherapy is known to enhance the sensitivity of tissues to radiation, thus contributing to the development of radiation-induced toxicities. The internal and external cohorts considered in this study differed largely in the percentage of patients receiving chemotherapy. The difference in chemotherapy rates between the ORN and control group was larger in the internal dataset, and the effect size of this variable was in opposite directions between datasets.

Our control-case matching approach used to address class imbalance was limited to primary tumour site and treatment year to ensure that the other variables could be used as confounders. This approach, however, introduced selection bias and affected the representation of tumor sites within our cohort. For example, only 3 % of our cases involved the larynx or hypopharynx, whereas in a population-based cohort studied by van Dijk et al. [9], this was 15 %. Van Dijk et al.'s cohort had a more diverse representation of low and high radiation dose levels in both ORN and control groups due to the higher proportion of larynx cases. The more pronounced diversity in radiation dose levels in their cohort, including both more low-dose early-stage larynx cases and higher-dose cases, likely contributed to better separation between ORN and non-ORN groups, provided more informative data for the model to learn from, enhancing the model's ability to discriminate between these groups. As a result, van Dijk et al.'s model showed better discrimination performance, reflected in a higher ROC AUC.

This study investigated the technicalities of translating DL-based toxicity prediction models to external datasets. Thus, a technically convenient class-balanced approach was used to develop and test the models, and both the internal and external datasets did not represent the real-world ORN prevalence. When a DL model trained on a class-balanced dataset is applied to a real-world population with different class distributions, the raw predicted scores may become poorly calibrated and should not be considered as actual ORN risk predictions. By recalibrating the model on real-world data distributions, the model's predicted probabilities could be aligned more closely with actual ORN risks, thereby enhancing its applicability and reliability in diverse clinical settings. In traditional NTCP models, model adjustments can often be done with methods like logistic regression on the entire dataset [20], [21], [22]. In DL models, however, a separate calibration dataset (ideally with a real-world class distribution) is required to apply methods like Platt scaling or Isotonic regression to map the raw scores to calibrated probabilities.

A number of measures were implemented to address the challenge of a small training dataset, including data augmentation, cross-validation resampling and dropout regularization. We also simplified the standard DenseNet-121 to a shallower and less complex DenseNet-40 CNN. However, while strong regularization measures helped mitigate overfitting, they could have affected model performance. On the other hand, further architecture simplification to reduce the number of model parameters could contribute to less overfitting, particularly in the multimodality model, which resulted in additional parameters when incorporating the clinical variables.

As is typically done in image processing, we performed normalisation of dose maps, thus removing information about absolute dose. However, this normalisation step should be reconsidered when information about absolute dose is required such as in post-hoc interpretability analysis for dosimetric association studies.

While we acknowledge other multimodality data fusion strategies [16], [17], a Joint Fusion approach was considered more suitable for NTCP modelling as it captures both modality-specific and cross-modal patterns during training, thus allowing to model the interaction between dosimetric and clinical variables.

Finally, in this study, the endpoint was simplified to a binary outcome (ORN vs. no ORN), which resulted in the loss of clinically valuable information regarding the severity of this toxicity. This decision was driven by the small dataset size, leading to an even greater underrepresentation of the various ORN stages. While a larger dataset (e.g., PREDMORN consortium [30]) would allow for addressing this limitation, the use of multiple ORN classification systems across institutions [31] remains a significant challenge that needs to be addressed.

Using radiation dose distributions of the mandible rather than DVH data, DL-based ORN NTCP models have the potential to leverage the inherent spatial information embedded in dose maps to enable post-hoc spatial localisation analyses. Consequently, the model outputs not only yield predicted toxicity probabilities but could also offer insights into spatial ORN risk, aiding in dental and oncological decision-making. While our study has demonstrated the potential of DL in ORN NTCP modelling, it has also underscored the risks of model overfitting and the criticality of addressing domain shift in multimodality DL-based NTCP models for accurate ORN prediction. Future efforts must focus on refining and validating spatial ORN models to ensure their clinical applicability for individual risk assessments.

## Author contributions

LHV, APK and TGU conceptualized and designed the study and methodology. LHV and VP performed the data collection and curation of the internal dataset and formal analysis, with input from VP, AK and TGU. CRH and JJ performed the data collection and curation of the external dataset. LHV drafted the original manuscript draft, including figures and tables, and all authors contributed to revising and editing the text. All authors reviewed and approved the final version of the manuscript.

## Declaration of competing interest

The authors declare that they have no known competing financial interests or personal relationships that could have appeared to influence the work reported in this paper.
