# Supplementary material for: External validation of a multimodality deep-learning normal tissue complication probability model for mandibular osteoradionecrosis trained on 3D radiation distribution maps and clinical variables
Source: Phys Imaging Radiat Oncol. 2024 Nov 2;32:100668. doi: 10.1016/j.phro.2024.100668 (PMC11574792; doi:10.1016/j.phro.2024.100668)
Supplement: Supplementary Data 1 [file mmc1.pdf]

Supplement A. Inclusion/exclusion criteria

The inclusion and exclusion criteria from the PREDMORN study protocol [31] were followed For the GSTT cohort, a total of 1721 HNC patients radically treated during the time span considered. From these, a total of 142 patients (8.3%) were diagnosed with ORN, 50 of which were excluded because of unavailable RT dose and/or RT plan files (18), ORN region outside of the mandible (15), palliative or low prescribed dose (8), previous irradiation in the HN region (6) or two primary tumour sites (3).

Table A1: Inclusion and exclusion criteria for the study.

**Inclusion criteria**	• HNC cases treated radically with RT or chemo-radiation (CRT) and post-operative RT (PORT)+/- chemotherapy (C-PORT) using IMRT or VMAT.<br>*Specific to ORN cases:*<br>• Confirmed diagnosis of ORN (any grade). ORN defined clinically as 'an area of exposed bone in the mandible that had been present for at least 8 weeks in a previously irradiated field, in the absence of recurrent tumour' [20]. NB: cases with recurrences outside of the HN region will be included.<br>*Specific to control cases:*<br>• Any histology except for T1/2N0 Larynx cases, as these do not receive significant dose to the mandible.<br>• The selection of controls should be as per the methodology described in Figure 1.
**Exclusion criteria**	• Datasets without available CT volume, RT structure or RT dose DICOM files.<br>• ORN outside the mandible (e.g. maxilla). NB: if multiple ORN sites, cases will be included if at least one of the sites is the mandible.<br>• Cases with re-irradiation to the HN region.<br>• Cases treated with less than a radical dose of radiation and/or those with life expectance less than 12 months.

Supplement B. Monaco vs. Eclipse TPS dose calculation differences

At GSTT, HNC treatment plans until 2016 were produced using the Monaco TPS (Elekta AB, Stockholm, Sweden), which uses a Monte Carlo (MC)-based dose calculation algorithm. Afterwards, the Eclipse TPS (Varian Medical Systems, Inc. Palo Alto, CA, US) with the Analytical Anisotropic Algorithm (AAA) was used instead.

MC dose calculation methods are highly accurate but very computationally expensive; they randomly simulate the interaction histories of a large number of particles through the treatment head and as they enter the different tissue types. The AAA is a kernel-based 3D pencil beam convolution-superposition algorithm. A dose spread kernel is a representation of the energy spread from photons and electrons resulting from the interaction of the primary photons in tissue at a given point. The AAA convolves the kernel with the fluence of energy transferred from the primary radiation beam to the secondary particles to calculate the absorbed dose.

Absorbed dose can be reported by the TPS as Dw,w, Dm,m or Dw,m, where the first subscript refers to the medium in which radiation transport occurs and the second subscript refers to the medium in which the energy is deposited. At GSTT, Dw,m was historically the choice for clinical treatment plans with the Monaco TPS. Dm,m was later adopted and all clinical treatment plans created with the Eclipse TPS have been calculated in Dm,m. Systematic differences of up to 2.7% between Dm,m and Dw,m have been previously reported [C1] for critical structures in the head and neck region. For the work described in this thesis, al Monaco plans originally created in Dw,m were recalculated into Dm,m.

[B1] Dogan N, Siebers JV, Keall PJ. Clinical comparison of head and neck and prostate IMRT plans using absorbed dose to medium and absorbed dose to water. Physics in Medicine and Biology, 51:4967–4980, 10 2006.

Supplement C. TRIPOD + AI checklist

TRIPOD+AI

Version: 11-January-2024

Section/Topic	Item	Development / evaluation[1]	Checklist item	Reported on page
**TITLE**				
Title	1	D;E	Identify the study as developing or evaluating the performance of a multivariable prediction model, the target population, and the outcome to be predicted	1
**ABSTRACT**				
Abstract	2	D;E	See TRIPOD+AI for Abstracts checklist	
**INTRODUCTION**				
Background	3a	D;E	Explain the healthcare context (including whether diagnostic or prognostic) and rationale for developing or evaluating the prediction model, including references to existing models	3
	3b	D;E	Describe the target population and the intended purpose of the prediction model in the context of the care pathway, including its intended users (e.g., healthcare professionals, patients, public)	3
	3c	D;E	Describe any known health inequalities between sociodemographic groups	
Objectives	4	D;E	Specify the study objectives, including whether the study describes the development or validation of a prediction model (or both)	3
**METHODS**				
Data	5a	D;E	Describe the sources of data separately for the development and evaluation datasets (e.g., randomised trial, cohort, routine care or registry data), the rationale for using these data, and representativeness of the data	4
	5b	D;E	Specify the dates of the collected participant data, including start and end of participant accrual; and, if applicable, end of follow-up	4
Participants	6a	D;E	Specify key elements of the study setting (e.g., primary care, secondary care, general population) including the number and location of centres	4
	6b	D;E	Describe the eligibility criteria for study participants	4
	6c	D;E	Give details of any treatments received, and how they were handled during model development or evaluation, if relevant	4
Data preparation	7	D;E	Describe any data pre-processing and quality checking, including whether this was similar across relevant sociodemographic groups	5
Outcome	8a	D;E	Clearly define the outcome that is being predicted and the time horizon, including how and when assessed, the rationale for choosing this outcome, and whether the method of outcome assessment is consistent across sociodemographic groups	5
	8b	D;E	If outcome assessment requires subjective interpretation, describe the qualifications and demographic characteristics of the outcome assessors	
	8c	D;E	Report any actions to blind assessment of the outcome to be predicted	
Predictors	9a	D	Describe the choice of initial predictors (e.g., literature, previous models, all available predictors) and any pre-selection of predictors before model building	5
	9b	D;E	Clearly define all predictors, including how and when they were measured (and any actions to blind assessment of predictors for the outcome and other predictors)	4
	9c	D;E	If predictor measurement requires subjective interpretation, describe the qualifications and demographic characteristics of the predictor assessors	
Sample size	10	D;E	Explain how the study size was arrived at (separately for development and evaluation), and justify that the study size was sufficient to answer the research question. Include details of any sample size calculation	4
Missing data	11	D;E	Describe how missing data were handled. Provide reasons for omitting any data	
Analytical methods	12a	D	Describe how the data were used (e.g., for development and evaluation of model performance) in the analysis, including whether the data were partitioned, considering any sample size requirements	5
	12b	D	Depending on the type of model, describe how predictors were handled in the analyses (functional form, rescaling, transformation, or any standardisation).	5
	12c	D	Specify the type of model, rationale[2], all model-building steps, including any hyperparameter tuning, and method for internal validation	5
	12d	D;E	Describe if and how any heterogeneity in estimates of model parameter values and model performance was handled and quantified across clusters (e.g., hospitals, countries). See TRIPOD-Cluster for additional considerations[3]	
	12e	D;E	Specify all measures and plots used (and their rationale) to evaluate model performance (e.g., discrimination, calibration, clinical utility) and, if relevant, to compare multiple models	6
	12f	E	Describe any model updating (e.g., recalibration) arising from the model evaluation, either overall or for particular sociodemographic groups or settings	
	12g	E	For model evaluation, describe how the model predictions were calculated (e.g., formula, code, object, application programming interface)	5
Class imbalance	13	D;E	If class imbalance methods were used, state why and how this was done, and any subsequent methods to recalibrate the model or the model predictions	
Fairness	14	D;E	Describe any approaches that were used to address model fairness and their rationale	
Model output	15	D	Specify the output of the prediction model (e.g., probabilities, classification). Provide details and rationale for any classification and how the thresholds were identified	6

[1] D=items relevant only to the development of a prediction model; E=items relating solely to the evaluation of a prediction model; D;E=items applicable to both the development and evaluation of a prediction model

[2] Separately for all model building approaches.

[3] TRIPOD-Cluster is a checklist of reporting recommendations for studies developing or validating models that explicitly account for clustering or explore heterogeneity in model performance (eg, at different hospitals or centres). Debray et al, BMJ 2023; 380: e071018 [DOI: 10.1136/bmj-2022-071018]

Training versus evaluation	16	D;E	Identify any differences between the development and evaluation data in healthcare setting, eligibility criteria, outcome, and predictors	
Ethical approval	17	D;E	Name the institutional research board or ethics committee that approved the study and describe the participant-informed consent or the ethics committee waiver of informed consent	4
**OPEN SCIENCE**				
Funding	18a	D;E	Give the source of funding and the role of the funders for the present study	1
Conflicts of interest	18b	D;E	Declare any conflicts of interest and financial disclosures for all authors	
Protocol	18c	D;E	Indicate where the study protocol can be accessed or state that a protocol was not prepared	Suppl. Data
Registration	18d	D;E	Provide registration information for the study, including register name and registration number, or state that the study was not registered	
Data sharing	18e	D;E	Provide details of the availability of the study data	
Code sharing	18f	D;E	Provide details of the availability of the analytical code[4]	5
**PATIENT & PUBLIC INVOLVEMENT**				
Patient & Public Involvement	19	D;E	Provide details of any patient and public involvement during the design, conduct, reporting, interpretation, or dissemination of the study or state no involvement.	
**RESULTS**				
Participants	20a	D;E	Describe the flow of participants through the study, including the number of participants with and without the outcome and, if applicable, a summary of the follow-up time. A diagram may be helpful.	Suppl. Data
	20b	D;E	Report the characteristics overall and, where applicable, for each data source or setting, including the key dates, key predictors (including demographics), treatments received, sample size, number of outcome events, follow-up time, and amount of missing data. A table may be helpful. Report any differences across key demographic groups.	8
	20c	E	For model evaluation, show a comparison with the development data of the distribution of important predictors (demographics, predictors, and outcome).	Suppl. Data
Model development	21	D;E	Specify the number of participants and outcome events in each analysis (e.g., for model development, hyperparameter tuning, model evaluation)	8
Model specification	22	D	Provide details of the full prediction model (e.g., formula, code, object, application programming interface) to allow predictions in new individuals and to enable third-party evaluation and implementation, including any restrictions to access or re-use (e.g., freely available, proprietary)[5]	5
Model performance	23a	D;E	Report model performance estimates with confidence intervals, including for any key subgroups (e.g., sociodemographic). Consider plots to aid presentation.	9
	23b	D;E	If examined, report results of any heterogeneity in model performance across clusters. See TRIPOD Cluster for additional details[3].	
Model updating	24	E	Report the results from any model updating, including the updated model and subsequent performance	
**DISCUSSION**				
Interpretation	25	D;E	Give an overall interpretation of the main results, including issues of fairness in the context of the objectives and previous studies	
Limitations	26	D;E	Discuss any limitations of the study (such as a non-representative sample, sample size, overfitting, missing data) and their effects on any biases, statistical uncertainty, and generalizability	10
Usability of the model in the context of current care	27a	D	Describe how poor quality or unavailable input data (e.g., predictor values) should be assessed and handled when implementing the prediction model	10
	27b	D	Specify whether users will be required to interact in the handling of the input data or use of the model, and what level of expertise is required of users	
	27c	D;E	Discuss any next steps for future research, with a specific view to applicability and generalizability of the model	10

From: Collins GS, Moons KGM, Dhiman P, et al. *BMJ* 2024;385:e078378. doi:10.1136/bmj-2023-078378

[4] This relates to the analysis code, for example, any data cleaning, feature engineering, model building, evaluation.
[5] This relates to the code to implement the model to get estimates of risk for a new individual.

Supplement D. Receiver operating characteristic curves

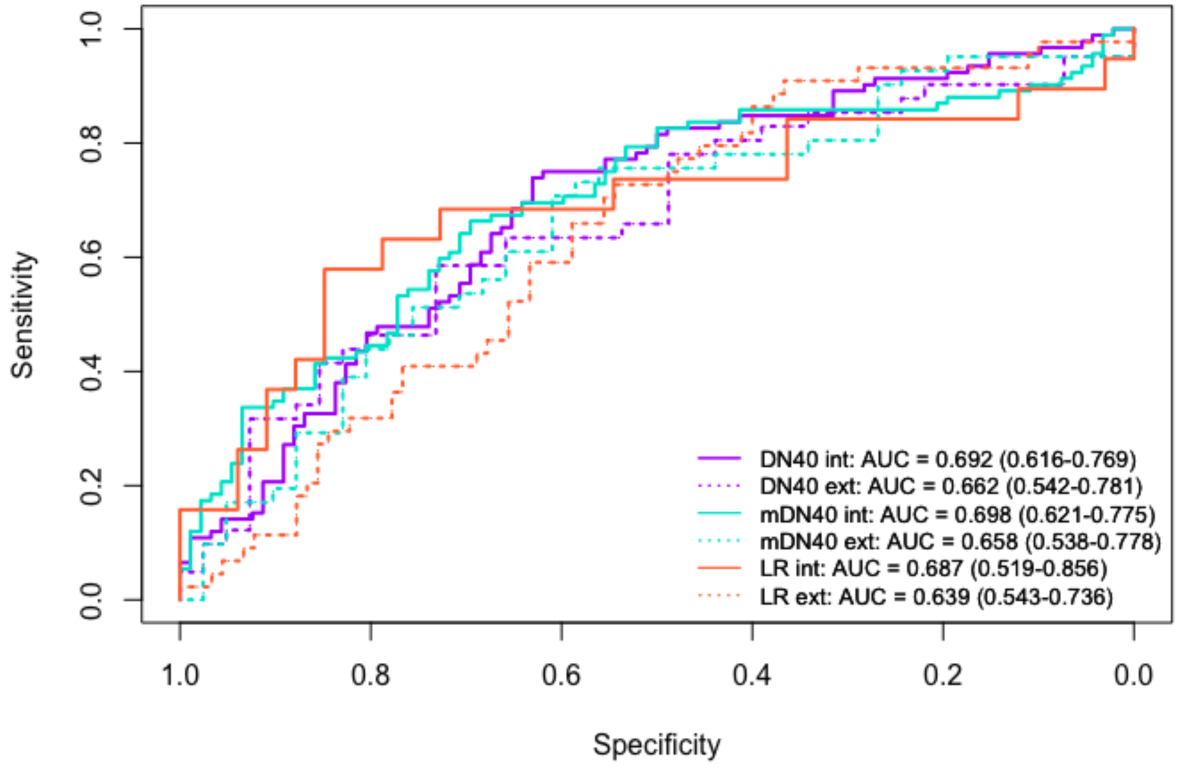

Figure D1. Receiver operating characteristic (ROC) curves of the 3D-DN40 (purple), 3D-mDN40 (blue) and LR (red) models at internal (continuous line) and external (dotted line) validation. The 3D-DN40 model was trained on 3D dose distribution maps only, the 3D-mDN40 model was trained on 3D dose distribution maps and clinical variables and the LR model was trained on DVH metrics and clinical variables.

Supplement E. Dosimetric comparison

While the models were trained on radiation dose distribution maps rather than DVH data, visual representation of the mean DVH for the internal and external datasets for each group (ORN and control) allowed for an overall assessment of dosimetric differences between groups and datasets**.** Although the dose distribution varied from patient to patient within each institution (largely depending on the primary treatment site), the dose distribution in the internal dataset (average Dmean of 45.3 Gy and 43.2 Gy for control and ORN groups) was overall higher than in the external dataset (average Dmean of 33.3 Gy and 32.2 Gy for control and ORN groups). The internal control group received similar intermediate doses to the mandible to the external ORN group, e.g., V40 Gy of 59.8% vs. 58.4% (figure 3).

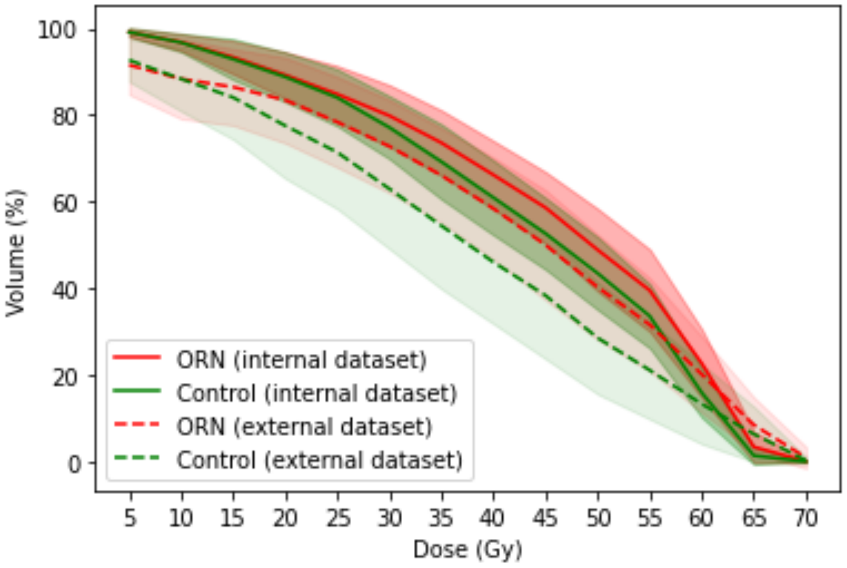

Figure E1. Mean dose-volume data comparison between the internal (GSTT) and external (OUH) datasets for the ORN and control groups separately. Doses are corrected to EQD2 considering an alpha-beta ratio of 3 for late effects. The shaded areas correspond to the 95% confidence intervals, where the darker and lighter shades correspond to the area between the upper and lower CI of the internal and external datasets, respectively.
